# Neuropsychological and behavioral disorders, functional outcomes and quality of life in traumatic brain injury victims

**DOI:** 10.11604/pamj.2021.38.346.16120

**Published:** 2021-04-11

**Authors:** Ghroubi Sameh, Feki Islem, Alila Samar, Chelly Hedi, Bouaziz Mounir, Elleuch Mohamed Habib

**Affiliations:** 1 Physical Medicine and Rehabilitation Department, University Hospital Center Habib Bourguiba, Sfax, Tunisia,; 2 Reanimation Department, University Hospital Center Habib Bourguiba, Sfax, Tunisia

**Keywords:** Traumatic brain injury, neuropsychological disorders, behavioral disorders, functional outcomes, patients

## Abstract

**Introduction:**

the assessment of neuropsychological and behavioral disorders outcomes, functional outcomes and quality of life in traumatic brain injury victims. It was also to evaluate initial means of care provided to these patients. Finally, to study correlations between neuropsychological and behavioral disorders with demographic characteristics, injury severity, functional status and quality of life.

**Methods:**

it was a cross-sectional study including 50 patients with traumatic brain injury conducted in the physical medicine and rehabilitation department of Sfax. Memory disorders were tested by the mini mental state and the Glaveston orientation and amnesia tests. Executive functions were evaluated by the dysexecutive function scale. The psychological profile was evaluated using the hospital anxiety and depression scale and behavioral disorders were tested by the agitated behavior scale. Glasgow outcome scale has allowed the assessment of traumatic brain injury severity in terms of disability. Otherwise, functional capacity was measured by functional independence measure scale. Finally, health-related quality of life was measured using a generic measure (short-form-36) and the QOLIBRI scales.

**Results:**

abnormal executive functions were noted in 41 patients (82%) with a dysexcutive function average score of 33.20 ± 22.74. About psychological profile, depressive symptoms were found in 32 patients (64%). Moreover anxiety was noted in 20 patients (40%). Behavioral disorders such as aggressiveness and agitation were noted respectively in 32 (64%) and 8 patients (16%). The global social functional evolution was considered as unfavorable in 42% of the patients and favorable in 58%. Regarding to functional independence measure scale, 92% of the victims showed impairment. Memory impairment and abnormal executive functions were statistically correlated with traumatic brain injury severity. Elementary brain injury lesions shown on computed tomography were correlated with memory disorders especially for temporal, cortical brain contusion and diffuse axonal injury. Our study showed that patients with severe memory impairment, abnormal executive functions and depressive mood had significant functional.

**Conclusion:**

the executive function disorders, depressed mood and the memory disorders seemed to be the most frequent among neuropsychological disorders in traumatic brain injury. We noted that it is so important to evaluate neuropsychological disorders in traumatic brain injury because they were underestimated. We have already started this experience despite the lack of means in our department. The evaluation of the executive function in addition to the classic neuropsychological assessment is essential to propose efficient means of rehabilitation.

## Introduction

Traumatic brain injury (TBI) is a leading cause of death and disability worldwide. A proportion of severe TBI survivors, after prolonged hospital care, require long rehabilitation and may have long-term physical, cognitive, emotional and behavioral disorders [1]. Although most of the TBI patients recover well from motor disorder, but they keep severe neuropsychological and behavioral problems months or years after the original head trauma often called invisible disability [2]. All of those symptoms may affect every aspect of an individual´s life, social, family relationships, professional rehabilitation and even the quality of life [3]. Their diagnosis is relatively simple in severe forms especially in patients with massive cognitive deficits. However, in patients with apparent good recovery, these disorders can be underestimated and go unnoticed if they are not evaluated by specialized teams [4]. Therefore it is of great importance to identify those at risk for poorer long-term outcomes. An adequate assessment of TBI must consider not only the results of neurotraumatic examination but also neuropsychological assessment with their social and professional repercussions. This will be very useful for the implementation of specific therapeutics and rehabilitative programs [5].

Literature analysis is complicated by the fact that the evaluation of these disorders is not always standardized [4]. Adolescents and adults surviving moderate and severe TBI often experience long-lasting complicated neuropsychological disorders including memory disorders, executive dysfunction, psychiatric disorders such as depression, anxiety, substance abuse, personality problems, and behavioral changes such as aggression and agitation [6]. It is generally considered that the recovery of cognitive, emotional and behavioral sequelae is carried out in the first six months [7] and that after one or two years their condition is stabilized. However, there is a lack of accurate information on the long-term neuropsychological outcome as well as its impact on social and occupational reintegration because few detailed and specific studies have been devoted to them. In fact, most of the studies devoted to this subject have an average of one to two years. More than five to ten years, only the patient´s family testament is available [8]. Data collection from the patient himself using standardized and detailed evaluation tests still very rare.

**The goals of our study were:** the assessment of neuropsychological and behavioral disorders outcomes, functional outcomes and quality of life in Tunisian TBI victims. It was also to evaluate our initial means of care and to compare them with those used worldwide to identify insufficiency and to perform better care. Finally, to study correlations between: neuropsychological and behavioral disorders with demographic characteristics, injury severity, functional status and quality of life.

## Methods

**Procedure:** it was a cross-sectional study including 50 patients with moderate or severe TBI conducted in the physical medicine and rehabilitation department between January and July 2016. All patients were contacted by telephone. A minority was already followed in our department. We have established a data sheet for each patient. We collected through it; epidemiological data, clinical data, initial orientation post resuscitation, neuropsychological, behavioral and functional outcomes. Informations (Glasgow Coma scale, coma length, radiological aspects) were collected from reanimation folders. The data sheet was fulfilled by the physician himself. The patient was called to the average of 2 to 3 times in order to properly pass the large number of scales.

**Participants:** for the present study, inclusion criteria were admission to a hospital for moderate (Glasgow Coma Scale (GCS) score, 9-12) or severe (GCS score, 3-8) TBI caused by a nonpenetrating trauma. Exclusion criteria were patients with aphasia, important pretraumatic neurologic, oncologic, or systemic impairments (spinal cord injury, psychiatric disorder, or cancer) that may interfere with TBI-related assessment of disability. All patients signed approved informed consent before entry into the study.

**Injury severity:** traumatic brain injury severity was assessed clinically by the Glasgow Coma Scale (GCS) before intubation and coma length. All of the patients with TBI underwent brain computing tomography scan immediately after the trauma as part of the standard clinical evaluation. The nature, extent, and location of the traumatic lesions were classified on the basis of the traumatic coma data bank criteria (Marshall *et al*. 1983) [9]. Magnetic resonance imaging was not performed to all patients. In fact, it has been done in 14 patients; those who had delayed awakening post artificial ventilation or patients which computed tomography (CT) scan results needs further examination by MRI.

**Neuropsychological and behavioral disorders outcomes:** cognitive, psychological and behavioral outcomes were evaluated. Memory disorders were performed by the Mini Mental State Examination (MMSE) [10] and the Glaveston Orientation and Amnesia (GOAT) [11] tests. Executive functions were evaluated by the dysexecutive function scale (DEX) [12]. The psychological profile was evaluated using the Hospital Anxiety and Depression Scale (HAD) [13] and behavioral disorders were tested by the Agitated Behavior Scale (ABS) [14].

**Functional outcomes:** Glasgow Outcome Scale (GOS) [15] has allowed the assessment of TBI severity in terms of disability. Otherwise, functional capacity was measured by Functional Independence Measure scale (FIM) [16].

**Quality of life:** health-related quality of life was measured using a generic measure (short-form-36) and the QOLIBRI. The QOLIBRI scale compromises six items with one item selected from each domain of QOLIBRI (physical condition, cognition, emotions, function in daily life, personal and social life, and current situation and future prospects) [17]. Responses to each item were scored 1 ("not at all") to 5 ("very"), and the sum of all items was converted to a percentage, with 0% representing the lowest score and 100% the best score. The short-form 36 measures the following eight dimensions of health: physical functioning (PF), role physical limitations (RP), bodily pain (BP), general health (GH), vitality (VT), social functioning (SF), role emotional limitations (RE) and mental health (MH). All eight SF36 scales are standardized to range from 0 to 100 with the higher score indicating better health status. An overall score under 66, 6 indicate that quality of life is impaired. In our study, we used a validated Arabic version of SF36 scale [18].

**Statistical analysis:** data were analyzed using SPSS Statistics 21.0 for windows. Demographic data were presented as mean ± SD. Correlations between neuropsychological and behavioral disorders with epidemiological and clinical characteristics, functional outcomes and the quality of life were studied with Pearson´s rank correlation coefficient. Prognostic ability of coma length and Glasgow coma scale were also evaluated with the area under the receiver operating characteristic (ROC) curve. Cut-off values for the prediction of dichotomized outcomes were defined using the ROC curve. A p-value < 0.05 was considered statistically significant. Area under the curve of 0.8-1.0 was considered very well, area under the curve of 0.7-0.8 adequate, and area under the curve < 0.7 poor [19].

## Results

**Epidemiological and clinical characteristic of the TBI patients:** information on demographics and clinical characteristic of the sample was shown in Table 1. A total of 50 patients including 40 (80%) male and 10 (20%) female were enrolled with an average age of 32.19 ± 12.37. Patients were reviewed with an average follow up of 4 years. Regarding with etiology of head trauma, motor vehicle accidents were the most frequent in TBI patients (84%). Referring to the Glasgow coma scale (GCS) in patients during admission, 66% had a severe accident with a score < 8. The mean ± SD of coma length and days of hospitalization in intensive care unit were 13.98 ± 13.16 and 33.61 ± 13.16 days respectively. In the Marshall classification according to computed tomography Scan findings, 58% of patients were in diffuse injury II class which corresponds to clear lesions in the scan or which have a mass effect and a midline shift < 5.

**Table 1:** demographic and clinical characteristic of the TBI patients

Characteristics	TBI, n = 50
Age, n (%)	32.19±12.37
[15-35]	30(60%)
[36-45]	16(32%)
≥46	4(8%)
Gender, n (%)	
Male	40(80%)
Female	10(20%)
Education level, n (%)	
Primary	26(52%)
Secondary	18(36%)
High school	6(12%)
Employment status, n (%)	
Professional activity	36(72%)
Primary level or student	9(18%)
Unemployed	5(10%)
Injury mechanism, n (%)	
Motor vehicle accident	42(84%)
Domestic accident	1(2%)
Aggression	3(6%)
Other	4(8%)
Injury severity (GCS), n (%)	
GSC 8-12	17(34%)
GSC <8	33(66%
Coma length	13.98±13.16 days
Days in ICU	33.61±13.16 days
Time since injury, n (%)	
<1years	2(4%)
1 to <2 years	5(1%)
2 to <4 years	8(16%)
4-15 years	35(70%)
Average follow up (years)	4
CT scan, n (%)	
Cerebral contusion	29(58%)
Subarachnoid hemorrhage	28(56%)
Subdural hematoma	19(38%)
Diffuse axonal lesions	18(36%)
Extradural hematoma	16(32%)
Marshall score, n (%)	
Class I	3(6%)
Class II	30(60%)
Class III	1(2%)
Class IV	13(26%)
Class V	3(6%)
MRI results: lesions location, n (%)	
Corpus callosum	8(16%)
White matter	6(12%)
Brainstem	5(10%)
Basal ganglia	3(6%)
Internal/external capsule	3(6%)
Thalamus	2(4%)
Cerebellum	1(2%)

**TBI**: traumatic brain injury, **GCS**: Glasgow coma scale, **ICU**: intensive care unit, **CT**: computing tomography, **MRI**: magnetic resonance imaging

**Initial management and orientation post resuscitation characteristics:** in our series, we found that only 2 patients (4%) were admitted in a private physical medicine and rehabilitation structure; there was no hospitalization in the physical medicine and rehabilitation department of Sfax which does not yet include any hospitalization bed. The remaining 96% returned to their homes directly after Intensive care unit hospitalization. Initial investigations showed that only 36% of these patients had an ambulatory follow up in our physical medicine and rehabilitation department. Their management consisted in botulism toxin injections (55%), physiotherapy (70%), ergotherapy (14%) and orthophony (11%). Otherwise, 60% escaped from any assessment and have been reintegrated into their families without follow-up. It should be noted that none of these patients have had a neuropsychological assessment.

**Neuropsychological and behavioral disorders outcomes:** Table 2 shows neuropsychological and behavioral disorders of the sample. Memory disorders were observed in 24 patients (48%) when evaluated by the mini mental state scale and in 17 patients (34%) by the Glaveston orientation and amnesia scale. Abnormal executive functions were noted in 41 patients (82%) with a dysexecutive function average score of 33.20 ± 22.74. The most affected dimensions were intentionality and negative effect. About psychological profile, depressive symptoms were found in 32 patients (64%). Moreover anxiety was noted in 20 patients (40%). Behavioral disorders such as aggressiveness and agitation were noted respectively in 32 (64%) and 8 patients (16%). Severe agitation level was found in one patient (2%).

**Table 2:** neuropsychological, behavioral, functional outcomes and quality of life

Scale	Value
MMS<23, n (%)	24(48%)
Mild: 20<MMS<23	11(45.8%)
Moderate:n10≤MMS≤20	8(33.3%)
Severe: MMS<10	5(20.8%)
GOAT≤75, n (%)	17(34%)
Moderate: 66≤GOAT≤ 75	10(58.8%)
Severe: GOAT<66	7 (41.1%)
Mean DEX score	33.20±22.74
Intentionality (%)	49%
Negative affect (%)	57%
Positive affect (%)	28%
Inhibition (%)	27%
Executive memory (%)	38%
HAD-D≥11, n (%)	32(64%)
HAD-A≥11, n (%)	20(40%)
ABS≥22, n (%)	8(16%)
Mild: 22≤ABS≤ 28	3(6%)
Moderate: 29≤ABS≤35	4(8%)
Severe: ABS≥ 35	1(2%)
GOS	
GOS: 1-2	29(58%)
GOS: 3-4	21(42%)
Mean FIM score	67.4±41.2
FIM = 18 (n, %)	3(6%)
FIM = 126(n, %)	4(8%)
Overall qolibri score (Mean, %)	46.05±21.76
Overall SF36 score	43.38±16.8 38(76%)
Overall score<66, 6 (n, %)	
PHS score	48.09±19.11
MHS score	38.67±17.71

**MMS**: mini mental state, **GOAT:** Galveston orientation and amnesia test, **DEX:** dysexecutive questionnaire, **ABS**: agitated behavior scale, **GOS**: Glasgow outcome scale, **FIM**: functional independence measure, **QOLIBRI**: finish quality of life after brain injury; **SF 36**: short form-36, **PHS**: physical health summary score, **MHS:** mental health summary score

**Functional outcomes:** according to the Glasgow outcome scale, the distribution is shown in Table 3. The global social functional evolution was considered as unfavorable (Glasgow outcome scale: 3-4) in 42% of the patients and favorable (Glasgow outcome scale: 1-2) in 58%. Regarding to functional independence measure scale, 92% of the victims showed impairment. Although often slight, at least one function has been affected. The functional independence measure scale ranged from 18 (6%) to 126 (8%) with a mean of 67.4 ± 4.

**Table 3:** correlations between injury severity (Glasgow coma scale and coma length), memory disorders and executive function

		GCS		P,R	Coma length	P,R
		GCS≤8 (N = 33)	GCS [9-12] (N = 17)			
MMS	MMS<23 (N = 24)	19	5	P = 0.043 R = 0.28	21.79±15.34	P<0.01 R = -0.43
	MMS>23 (N = 26)	14	12		6.77±3.25	
GOAT	GOAT≤75 (N = 17)	15	2	P<0.01 R = 0.41	24.41±16.34	P<0.01 R = -0.49
	GOAT>75 (N = 33)	18	15		8.61±6.66	
DEX		39.9±20.73	19.37±20.86	P<0.01 R = -0.40		P<0.01 R = 0.40

**MMS**: mini mental state, **GOAT:** Galveston orientation and amnesia, **DEX:** Dysexecutive questionnaire

**Quality of life:** about QOLIBRI scale, the overall score was of 46.05%. Patient satisfaction for thought, emotions, independence and social relations were respectively of 55.17%, 46.35%, 43.43% and 42.9%. The patients discomfort tested in relation to their feelings and their physical condition were respectively of 43.66% and 51.52%. About short-form 36 scale, the overall score was of 43.38. Impaired quality of life was observed in 76% of the patients. Table 2 showed score distributions for the short-form 36 subscales.

### Correlations study

**Figure 1:**
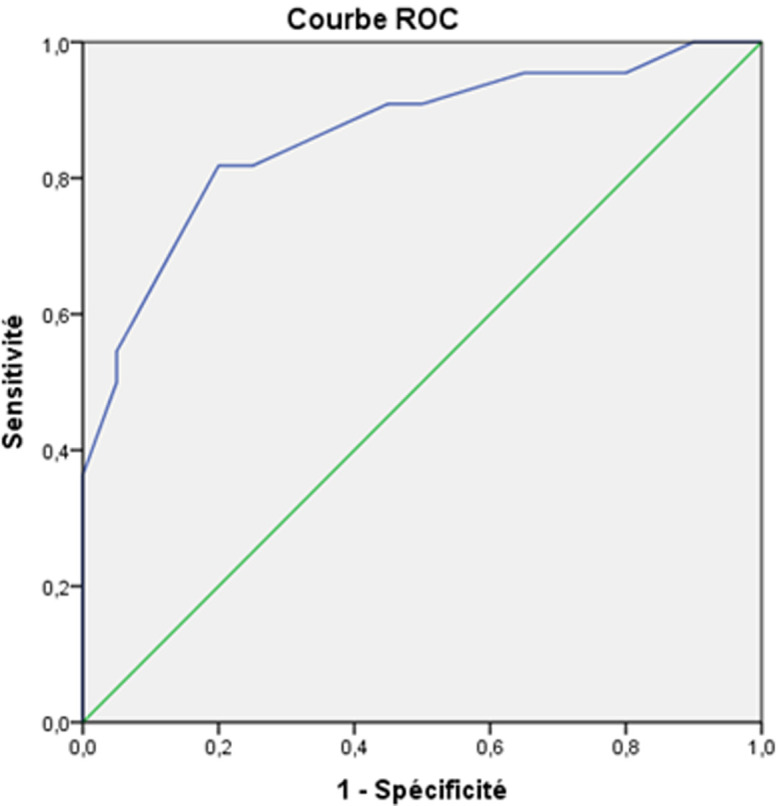
ROC curve showing the correlation between the coma length and MMS (AUC = 0.86; Se = 95%, Sp = 65%; p <0.01)

**Figure 2:**
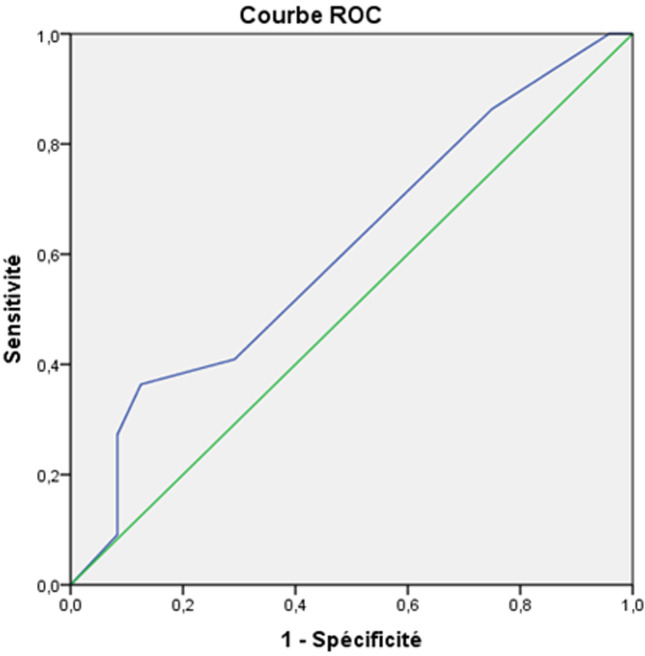
ROC curve showing the correlation between the Glasgow Coma Scale and MMS (AUC = 0.60; Se = 86%, Sp = 75%; p <0.01)

**Correlations between neuropsychological and behavioral disorders with demographic and clinical characteristics:** the study of correlations between neuropsychological, behavioral troubles and demographic, clinical variables showed that there is no correlation between memory disorders, executive functions, depressive mood and age. However, there was correlations between memory disorders and the education level (p = 0.047, r = 0.28), between aggression and male sex (p < 0.01, r = 0.7). Memory impairment and abnormal executive functions were statistically correlated with the TBI severity evaluated by initial Glasgow coma scale and the coma length (Table 3). However, neither psychological disorders such as depression, anxiety, nor behavior disorders such as agitation were correlated with the injury severity. Receiver operating characteristic analysis showed that the area under the ROC curve for coma length to help distinguish mini mental state <23 Vs. Mini mental state >23 was 0.86 (95%, CI 0.478-0.926). Area under the ROC curve for Glasgow coma scale to help distinguish mini mental state <23 Vs. Mini Mental State > 23 was 0.60 (95%, CI 0.444-0.773). Cut-off values for coma length and Glasgow coma scale were defined. For coma length, the cut-off value (mean) for memory impairment was 6.5 days with a sensitivity of 95% and specificity of 65%. For Glasgow coma scale, the cut-off value (mean) for memory impairment was 7.5 with a sensitivity of 86% and specificity of 75%. The ROC curves are displayed in Figure 1 and Figure 2.

**Correlations between neuropsychological and behavioral disorders with radiological aspects:** there were correlations between the elementary brain injury lesions shown on CT and memory disorders (MMS) especially for temporal (p = 0.039, r = 0.38), cortical brain contusion (p = 0.045, r = 0.31) and diffuse axonal injury (p = 0.047, r = 0.28). Furthermore, frontal contusions lesions were correlated with behavior disorders such as agitation (p = 0,046, r = 0.29). The highest dysexecutive function scores were associated with frontal contusions and diffuse axonal injury but there were not significant correlations. In our study, the extent of computed tomography lesions mentioned by the Marshall classification was not correlated with memory impairment severity, neither with the achievement of executive function nor with behavior disorders. Lobar white matter abnormalities shown in magnetic resonnance imaging were correlated with memory disorders with (p<0.01, r = 0.43).

**Correlations between neuropsychological and behavioral disorders with functional outcomes:** our study showed that patients with severe memory impairment, abnormal executive functions and depressive mood had significant functional dependence as well as a poor overall evolution with respective correlation coefficients shown on Table 4. A significant correlation was found between the functional status (FIM) and the overall evolution (GOS) (p< 0.01, r = -0.9).

**Table 4:** correlations between neuropsychological disorders, functional outcomes and quality of life

		GOS		P,R	FIM	P,R	QOL %	P,R	SF36		P,R
		GOS (1-2) (N = 29)	GOS(3-4) (N = 21)								
MMS	MMS<23 (N = 24)	8	16	P<0.01 R = -0.42	44.60±29.68	P<0.01 R = 0.43	43.5±21.2	NS	20	4	P<0.01 R = 0.48
	MMS>23 (N = 26)	21	5		92.17±37.47		44.2±23.1		18	8	
GOAT	GOAT≤75 (N = 17)	5	12	P<0.01 R = -0.48	42.58±26.02	P<0.01 R = 0.51	39.7±24.7	NS	15	2	P<0.01 R = -0.4
	GOAT>75 (N = 33)	24	9		82.48±40.3		41.5±22.9		23	10	
DEX		28.41±21.8	37.17±23.03	P<0.01 R = 0.52		P<0.01 R = -0.48		P = 0.045 R = -0.57			P = 0.034 R = -0.36
HAD	HAD-D≥11 (N = 32)	13	19	P = 0.03 R = 0.32	56.7±42.45	P = 0.017 R = -0.56	37.6±21.4	P<0.01 R = -0.4	26	6	P<0.01 R = -0.39
	HAD-D<11 (N = 18)	16	2		68.18±38.86		48.5±23.6		12	6	

**MMS**: mini mental state, **GOAT:** Galveston orientation and amnesia test, **DEX:** Dysexecutive questionnaire, **HAD**: hospital anxiety and depression, **GOS:** Glasgow outcome scale, **FIM:** functional independence measure, **QOL**: QOLIBRI, **SF36**: short- Form 36, **NS**: non significative

**Correlations between neuropsychological and behavioral disorders with quality of life:** depressed mood and abnormal executive function were significantly correlated with QOLIBRI scale (p<0.01, r = -0.4; p = 0.045, r = -0.57). There were no significant correlations between QOLIBRI and the other variables (age, sex, education level, injury severity, memory disorders). Otherwise, memory disorders, executive dysfunction and depressed mood were significantly correlated with the short-form 36 scale with respective correlation coefficients shown on Table 4.

**Correlations between quality of life and functional outcomes:** a significant correlation was found between the functional status measured by the the (functional independence measure scale) and the quality of life (P <0.01, r = 0.6). In fact patients who were more dependant (having the lowest functional independence measure score) had a poorer quality of life. The global social-functional evolution measured by Glasgow Outcome Scale was correlated to the QOLIBRI scale (P<0.01, r = -0.68) and to the (short form 36) scale (p<0.01, r = -0.64).

## Discussion

In our study, the assessment of TBI patients confirmed the persistence of cognitive, affective and behavioral difficulties 4 years after the TBI. The executive function disorders, the depressed mood and the memory disorders seemed to be the most frequent among neuropsychological disturbances. Memory and executive function impairment were correlated to injury severity evaluated by Glasgow coma scale, coma length and radiological factors (temporal, cortical lesions and diffuse axonal injuries). These disturbances, often underestimated, have a negative impact on the long term overall evolution and the quality of life. All patients have benefited for the first time of neuropsychological assessment and only thirty six percent of them had an ambulatory follow up in our polymyalgia rheumatica (PMR) department.

**Initial management and orientation post resuscitation:** none of our patients have benefited from cognitive assessment and only 36% of them having neuro orthopedic visible sequelae were addressed to our physical medicine and rehabilitation unit. The received care consists mainly of botulinism toxin injections and physiotherapy. This may be explained by the fact that there was no hospitalization bed in the physical medicine and rehabilitation public department of Sfax despite administrative requests since 1994. We noted also the lack of interest for cognitive impairment assessment in TBI patients in our practice due to the unavailability of specialized teams. So patients with apparent good recovery escaped from any neuropsychological assessment and are reintegrated into their families without follow-up. A study conducted in Tlemcen (Algeria) about a cohort of 377 severe TBI (in 2009 and 2010) found that only 10 patients were admitted to physical medicine and rehabilitation structure. This was due to the limited number of places [20]. Bosserelle [21] studied a cohort of 122 serious TBI in Ile de France (from 2005 to 2007) and found that hospitalization in a physical medicine and rehabilitation structure concerned 60% of these patients. The remaining 40% escape any assessment of cognitive impairment and didn´t have a follow up (35% return directly to their homes and 5% have an unknown trajectory). This discrepancy between the number of TBI and the number of visits to rehabilitation is reported by several studies, notably Bedjaoui [22]. Thus, the care pathways of TBI are quite random; it depends not only on the patient´s clinical condition but especially on the availability or not of beds in the physical medicine and rehabilitation structures. The management of the higher functions disorders (language, attention, memory...) is a real problem due to the unavailability of qualified personnel for this type of rehabilitation.

**Cognitive impairment:** there is growing evidence that a history of TBI places individuals at greater risk for cognitive decline across the life [23]. Among cognitive changes seen after TBI, it was found that memory disorders and executive function disorders had been predominant. In our study, memory disorders were frequently found after head trauma. They were one of the most frequent complaints of TBI. Prevalence of memory disorders vary greatly depending on the population group and series. This is due to the different means of assessment. In our series, memory disorders were maintained 4 years after TBI in 24 patients (48%) when evaluated by the mini mental state scale and in 17 patients (34%) by the Glaveston orientation and amnesia scale. This is likely due to the specificity of Glaveston orientation and amnesia test to evaluate memory disorders in TBI [24]. Mac Allister [25] reported a prevalence of 40 to 60% of memory and attention deficits 1-3 months after mild TBI. Levin [26] reported that 30-50% of severe TBI still had several memory impairment years after the head trauma. Executive dysfunction is among the most common and disabling aspects of cognitive impairment following TBI, and may include deficits in reasoning, planning, concept formation, mental flexibility. In our study; its frequency was even higher than memory disorders. The assessment of executive function disturbance is difficult due to the lack of sensitivity and specificity of traditional neuropsychological tests. Many frontal scales were used to evaluate dysexecutive syndrome such as bevioural assessment of the dysexecutive syndrome (BADS), Batterie Rapide d´Efficience Frontale (BREF). Due to the unavailability of theses batteries, dysexecutive function questionnaire was used in our study. The cognitive deficits vary depending on the severity of the injury. In our series, ROC analysis showed that coma length was a very good predictor of memory disorders. Patients who experienced more than 6.5 days of coma seemed to be more likely to develop memory disorders after a mean follow-up of 4 years of moderate to severe TBI. In contrast GCS was considered as a poor predictor of memory impairment (area under the ROC curve < 0.7).

The relationship between TBI severity and neuropsychological deficits has been studied up to 10 years after injury [27]. Most available research shows that individuals with uncomplicated, moderate TBI typically recover to baseline levels of cognitive functioning within 1 to 3 months after injury and are expected to have a favorable long-term outcome [28]. Prolonged recovery course has been associated with more severe acute injury indicators (e.g. Glasgow coma scale, coma length or initially more severe symptoms) [29]. Levin [26] reported that long lasting memory disorders depends on coma length. Patients, who experienced less than 10 days of coma, seemed to have returned to a normal level between the 12^th^ and 24^th^ month. Previous studies have consistently found an association between fronto-temporal damage typical of TBI and cognitive deficits [30]. In our study, the analysis of correlations between cognitive impairment and the elementary brain injury lesions showed that memory disorders were correlated to temporal, cortical brain contusion and traumatic axonal injury. Similar results were found in previous studies [31]. Besides, we found that lobar white matter abnormalities shown in magnetic resonnance imaging were correlated with memory disorders. This fact was demonstrated in a study using diffusing tensor imaging technique [32]: the moderate to severe TBI subjects demonstrated reduced white matter integrity, relative to controls.

It has been established that executive functions are sensitive primarily to damage of the frontal lobe, including dorsolateral, orbital, and medial structures of the prefrontal cortex [33]. Other studies [34,35] have shown that the severity of the executive functions disorders was more related to the extent of the diffuse axonal lesions than to the direct lesions of the frontal systems. In our study; diffuse axonal injuries and frontal contusions were associated to highest dysexecutive function scores but no statistical correlations were found. In our series, the extent of computed tomography lesions mentioned by the Marshall classification was not correlated with memory impairment severity, neither with the achievement of executive dysfunction. A similar result was found by Levin *et al*. [36]. Nevertheless, Mataró *et al*. reported that there was a relationship between the acute intracranial lesion diagnosis according to the Marshall computed tomography classification and neuropsychological results at 6 months post-injury [37]. Among epidemiological characteristics studied, cognitive troubles did not have a correlation to age or sex. Furthermore, these troubles were hard felt in patients with low level of education. These results are similar to those of Alaoui [38]. Otherwise, he found that problems in capacity to plan and concept disorganization were significantly higher in women.

**Psychiatric and behavioral disorders:** psychiatric disorders are common following TBI and including depression, anxiety, and psychosis, as well as other maladaptive behaviors and personality changes. The published rates of psychiatric disorders in patients with TBI are 14-77% for depression, 3-28% for generalized anxiety disorder and 2-17% for behavior disorders [39]. The main findings of our study are that depression was the most frequent psychiatric diagnosis observed in 64% of the patients after a mean follow up of 4 years. Our findings are in line with earlier reports of high rates of major depression after TBI [40]. MC Mauri *et al*. observed depression in 62.5% of the patients after one month, 50% after three months, and 43.75% after six months. Furthermore, depression has been observed not only soon after TBI, but also throughout a 30-year follow-up [41]. Like others [42], we found that the occurrence of psychiatric disorders was not significantly related to the severity of the brain injury. Although, further studies found a significant relationship between trauma severity and the presence of more severe psychiatric symptoms; thus demonstrating that the severity of TBI may cause a worsening of psychiatric symptomatology and of other neurobehavioral difficulties [25]. Behavioral disorders are part of the clinical spectrum of head trauma and are defined by visible reactions of a person in response to his environment. Significant changes in character, anger and aggression were observed in our study. Aggressiveness was a common symptom especially in males observed in 64% of patients. Agitation was less frequent, found in 16% of TBI in which 1 patient (2%), have had a severe agitation level according to the agitated behavioral scale. These behavior disorders may affect 50 to 70% of brain trauma victims [43].

**Functional outcomes:** the Glasgow outcome score was proposed in order to class patient´s outcome in terms of mortality, functionality, and economic reintegration. Bosserelle [21] found in his cohort (one year after a severe TBI): 2% in vegetative state, 38% Glasgow outcome scale 3.41% Glasgow outcome scale 2, and 19% had good recovery (Glasgow outcome scale: 1). According to an evaluation made 4 years after TBI, Darnoux [44] found that: 30% were Glasgow 0utcome scale 1.39% were Glasgow outcome scale 2 and 31% were Glasgow outcome scale 3. In our series, 4% of TBI were classified as Glasgow outcome scale 4.38% Glasgow outcome scale GOS 3.28% Glasgow outcome scale 2 and 30% had good recovery (Glasgow outcome scale 1). In our study, Correlations between different neuropsychological disorders and functional outcomes showed that patients who had memory impairment, abnormal executive functions and depressive mood were more dependent on the functional aspects and had a poor overall evolution. Several studies support the view that cognitive impairment functions affects long-term functional status after acute cerebral attacks such as TBI [45]. Cognitive disorders are the major cause of disability after TBI. Victims of TBI will be characterized mainly by a global slowdown with difficulties to learn, to concentrate, to show mental flexibility [38]. Attention and executive functions are the main causes of disability after head trauma and regularly lead to complaints from patients and families [46].

**Quality of life:** traumatic brain injury (TBI) affects many domains of life, and impacts the quality of life experienced by the injured person [47]. The QOLIBRI instrument was developed specifically for persons after TBI [48]. In recent years QOLIBRI has become an important outcome variable after TBI [49] alongside the more traditional outcome measures. In our study, the overage of patients was dissatisfied with their quality of life several years after the TBI. The most affected dimensions were feeling and social relations. Earlier studies have shown that QoL after TBI is linked to changes in emotional status [50], neurobehavioral disturbances and cognitive impairments [51]. In our study depression was found to be the strongest predictor of impaired quality of life (QOLBRI scale). This can be explained by the bad socio-economic conditions with a lack of human and material aids for therapeutic management, despite that the constant presence of the family members to provide moral and material support [52]. The strong association between the QOLIBRI and emotional state was expected on the basis of the theoretical model and the analysis of the international data and from previous research [53]. Functional outcome measured by GOS was significantly correlated to the QOLIBRI and to the Short Form 36. A similar finding has been reported in some previous research [40] and suggests adjustment to disability caused by TBI.

## Conclusion

In summary, a history of TBI was associated with increased risk of cognitive, affective and behavioral difficulties years after the original head trauma in Tunisian TBI patients. Executive dysfunction, depressed mood and memory disorders were the most frequent among neuropsychological disturbances. These disorders are a major source of disability, they have a negative impact on the long term overall evolution and even the quality of life. These neuropsychological disorders were underestimated in our population. In fact, none of our patients have benefited from cognitive assessment and only those having neuro orthopedic visible sequelae, were addressed to our physical medicine and rehabilitation department. In our physical and rehabilitation team we are convinced that is necessary to implement means of screening, and specific therapeutic and rehabilitation programs. We have already started this experience despite the lack of means in our department. Further studies with larger cohorts will be needed to better understand the pathophysiology of such troubles and to establish a better management.

### What is known about this topic

- Traumatic brain injury is a leading cause of death and disability worldwide;
- Although most of the traumatic brain injury patients recover well from motor disorder, but they keep severe neuropsychological and behavioral problems months or years after the original head trauma often called invisible disability.

### What this study adds

- An adequate assessment of traumatic brain injury patients must consider not only the results of neurotraumatic examination but also neuropsychological assessment with their social and professional repercussions;
- In our study we tried to evaluate and to understand the pathophysiology of such troubles. This will be very useful for the implementation of specific therapeutics and rehabilitative programs;
- We tried also to evaluate our initial means of care and to compare them with those used worldwide to identify insufficiency and to perform better care.
